# Advances in Sport and Performance Nutrition

**DOI:** 10.3390/nu11030538

**Published:** 2019-03-02

**Authors:** Antonio Paoli

**Affiliations:** 1 Department of Biomedical Sciences, University of Padova, 35131 Padova, Italy; antonio.paoli@unipd.it; 2 Faculty of Sport Sciences, UCAM, Catholic University of Murcia, 30107 Murcia, Spain; 3 European Sport Nutrition Society, 43126 Parma, Italy

This monograph “Advances in Sport and Performance Nutrition” collects 12 papers from several countries, from Australia to Thailand, of which 10 are original researches and two are reviews. This volume confirms the fact that sport nutrition is a wide and multifaceted field of investigation. One emerging topic in sport nutrition and also in general human nutrition is the “deep sea” of microbioma research. In this volume, Mortaza and colleagues investigate the effects of a period of intensified endurance training during a high-carbohydrate diet or very low-carbohydrate ketogenic diet. The effects of a ketogenic diet on sport performance is a controversial topic [1]. The results of Mortaza and colleagues suggest that a low-carbohydrate, high-fat ketogenic diet influences the relative abundances of some key bacterial taxa, with an increase of Bacteriodes that paradoxically correlates with fat oxidation [2]. The amount and the type of carbohydrates (i.e., low glycemic index versus high glycemic index [3]) in sport nutrition has been an important field of investigation in the last decades. The novelty of the paper of Vlahoyiannis and co-workers [4] is the focus on the effects of the high or low glycemic index of a post-exercise meal on another “hot” topic in sport and performance: the quality of sleep. They suggested that a high glycemic index meal, following a single spring interval training session, can improve both sleep duration and sleep efficiency, while reducing in parallel sleep onset latency. One of the oldest supplements used for the legal improvement of sport performance is sodium bicarbonate (NaHCO_3_). From the seminal paper of the Harvard Fatigue Laboratory [5], through the more NaHCO_3_-focused works of Jones and colleagues [6] to the very last papers published, the right dosage and timing of NaHCO_3_ supplementation and its combination with other molecules [7,8] is a very important topic for sport nutrition. The overall small but positive effects (2–3%) on different performance outcomes [9] vindicate the ever-present attention to this supplement.

One of the main issues for athletes and coaches is recovery. Often unrecognized, recovery is however a fundamental part of every training program. There are many factors affecting recovery [10], and appropriate nutrition is one of the most important. In this field, so-called “functional beverages” could play a role [11] together with other supplements, such as omega 3. It is well known that omega 3 plays a fundamental role in reducing physiological inflammation [12] through resolvins [13], and its utility for muscle damage and recovery in sports has been widely demonstrated [14]. Also, many other supplements have been advocated to be helpful for athletes’ recovery strategies (antioxidant-rich foods, creatine, curcumin, etc.). Above all, quercetin deserves more attention; for example, it has been demonstrated to exert a cardioprotective effect on mdx mice [15]. In this special issue, Bazzucchi and colleagues demonstrated that quercetin supplementation attenuates the severity of muscle weakness caused by eccentrically-induced muscle damage [16]. Also, protein intake is critical for muscle recovery after exercise. Protein supplementation is the most-used supplement among athletes and gym enthusiasts [17], and recently the daily protein need for athletes has been settled well above the RDA [18]. Interestingly, protein intake is critical also during weight loss in elite athletes [19], especially in weight category sports where rapid weight loss is often achieved through excessive caloric reduction [20]. However, many questions are still open about protein supplementation; for example, the optimal requirement for athletes with different lengths of training experience [21], timing of supplementation [22], or different types of protein [23]. Whilst the latter has been recently investigated, the different effects of essential amino acids (EAA) and whey protein on aminoacidemia are still not clear; in this respect, Nakayama and collaborators showed that whey protein can induce a higher level of aminoacidemia compared to an essential amino acid blend [24]. A few final words should be devoted to a new complex chapter in sport nutrition: the inter-individual differences in response to supplements. Recently, there has been a great debate about low, normal and high responders to training [25], both in endurance [26] and resistance training [27,28], but the same approach could be used for sport supplements. Some data about the lack of caffeine’s ergogenic effects [29] or about ethnic differences in the response to a New Zealand blackcurrant extract [30] may be explained by some genetic and/or microbiota differences among individuals. All things considered, sport nutrition is a growing, interesting field of research that deserves wider and more strictly-controlled studies in the future.
